# Clinical descriptive analysis of severe *Pneumocystis jirovecii* pneumonia in renal transplantation recipients

**DOI:** 10.1080/21655979.2021.1911203

**Published:** 2021-04-25

**Authors:** Dan Xie, Wen Xu, Jingya You, Xiaofeng Yuan, Mingliang Li, Xiaogang Bi, Kouxing Zhang, Heng Li, Ying Xian

**Affiliations:** a Department of General Intensive Care Unit, Lingnan Hospital, The Third Affiliated Hospital of Sun Yat-sen University, Guangzhou, People’s Republic of China; b Department of Kidney Transplantation, Lingnan Hospital, The Third Affiliated Hospital of Sun Yat-sen University, Guangzhou, People’s Republic of China

**Keywords:** Metagenomic next-generation sequencing (mNGS), *pneumocystis jirovecii* pneumonia (pjp), renal transplantation, opportunistic fungal infection, trimethoprim-sulfamethoxazole (tmp-smx)

## Abstract

*Pneumocystis jirovecii (P. jirovecii)* pneumonia (PJP) is an opportunistic fungal infection after renal transplantation, which is always severe, difficult to diagnose, combined with multiple complications and have poor prognosis. We retrospectively analyzed clinical data, including risk factors, diagnosis, treatment and complications of seven clinical cases suffered with severe PJP after renal transplantation in our department in 2019. All the seven recipients were routinely prescribed with PJP prophylaxis after renal transplantation, and six of them suffered acute graft rejection before the infection. *P. jirovecii* sequence was identified in blood or broncho-alveolar lavage fluid (BALF) by the metagenomic next-generation sequencing (mNGS) in all patients. All the patients were improved with the therapy trimethoprim-sulfamethoxazole (TMP-SMX) combined with caspofungin for the PJP treatment, but suffered with complications including renal insufficiency, leukopenia, thrombocytopenia, gastrointestinal bleeding, mediastinalemphysema, pulmonary hemorrhage, and hemophagocytic syndrome and other severe infections. Taken together, mNGS is a powerful tool that could be used to diagnose PJP in renal transplantation recipients. And PJP prophylaxis should be prescribed during and after treatment for acute rejection. TMP-SMX is the first-line and effective drug for PJP treatment, but the complications are always life-threatening and lead to poor prognosis. We should pay attention to these life-threatening complications.

## Background

Allogeneic renal transplantation (RT) is recognized as the optimal treatment for end-stage renal disease. *Pneumocystis jirovecii* (*P. jirovecii*) pneumonia (PJP) is a fatal opportunistic fungal infection after renal transplantation, even with routinely PJP prophylaxis [1,2]. It was associated with high rates of intubation and mortality in RT recipients, which is always severe, difficult to diagnose, combined with multiple complications and have poor prognosis [3,4]. PJP outbreaks have been reported in many countries, such as British [5], North American [6], France [7]. The clinical presentation generally includes fever, dyspnea with hypoxemia, and nonproductive cough. The diagnosis of PJP is based on direct immunofluorescent staining and quantitative nucleic acid amplification of respiratory specimens. At present, trimethoprim–sulfamethoxazole (TMP-SMX) is the first-line agent and drug of choice for therapy of the PJP [8]. However, the positive rate of immunofluorescent staining in the sputum or bronchoalveolar lavage fluid (BALF) is low, and the quantitative nucleic acid amplification of *P. jirovecii* is not available, resulting in poor therapeutic effect. Therefore, with the limitations of conventional diagnostic approaches, non-targeted metagenomic next generation sequencing (mNGS) has been increasingly applied to diagnosis of infectious diseases [9]. And mNGS can be directly executed on clinical specimens [9].

mNGS allows identification and genomic characterization of bacteria, fungi, parasites and viruses without directly obtaining prior knowledge of specific pathogens from clinical specimens [10]. Wilson et al. [11] used mNGS from 7 meningitis patients to identify parasites, viruses and fungi. In addition, Wang et al. [12] found that the sensitivity of mNGS to diagnose mixed lung infections was significantly higher than that of conventional tests. Even Chen et al. [13] collected BALF from a patient with acute pneumonia after kidney transplantation, and found a large number of *P. jirovecii* reads using mNGS diagnosis, but no *P. jirovecii* was found after stained sputum and BALF smears. Confirm the diagnostic value of mNGS for PCP. However, there are still few reports on the use of mNGS after the occurrence of PJP after kidney transplantation.

Therefore, we collected BALF from renal transplant recipients and used mNGS to identify the *P. jirovecii*. And we retrospectively descriptive analyzed the clinical data of the seven renal transplant recipients who developed PJP after routinely PJP prophylaxis post-transplantation.

## Materials and Methods

### Patients

We retrospectively descriptive analyzed clinical data of seven patients treated for severe PJP in the renal transplant recipients, who transferred to our department from department of kidney transplantation in 2019. The data of demographics, renal transplantation operation time, induction and maintenance of immunosuppressive agents, PJP prophylaxis and cytomegalovirus (CMV) prophylaxis, acute rejection events, anti-rejection therapy, and the blood concentrations of calcineurin inhibitor (CNI) immunosuppressant were collected.

## Clinical measurements

The blood routine examination, function of liver and kidney, plasma (1,3)-β- D-glucan test (BDG), plasma loads of CMV-DNA, and the bacterial and fungal cultures of blood, urine, sputum and BALF, chest computed tomography (CT) scan were retrospectively analyzed.

BALF samples were harvested when the patient received mechanical ventilation for the immunofluorescent staining and mNGS detection. The diagnostic test of choice is generally the induced sputum or BALF examination with direct immunofluorescent staining for *P. jirovecii*.

## mNGS and analysis

The mNGS was performed by Visionmedicals.com (Guangzhou, China). The procedure for BALF samples includes nucleic acid extraction, library construction, sequencing, and information analysis as previously described [14].

## Treatment

Anti-thymocyte globulin (ATG) or anti-human T lymphocyte porcine Immunoglobulin (ALG) or basiliximab was given as induction immunosuppressant. All patients received oral maintenance immunosuppressant after RT, including tacrolimus (Tac) or cyclosporine A (CsA), mycophenolic acid (MFA) and prednisone (Pred).

PJP prophylaxis post-transplantation was routinely prescribed with TMP-SMX (twice a week, once a day, two tablets (160/800 mg) each time, each tablet contains 80 mg trimethoprim and 0.4 g sulfamethoxazole) for 3 months, and CMV prophylaxis post-transplantation was routinely prescribed with ganciclovir (one tablet (0.45 g) a day) for 2 months.

The clinical symptoms, therapeutic regimen for PJP, oxygen therapy, the complications and the outcomes were also analyzed.

## Results

### Baseline characteristics

All seven patients received renal transplantation in end-stage renal disease from donation after Cardiac Death. There were two females and five males. The age ranged from 35 to 60 years. All patients were HIV negative. Before the occurrence of PJP infection, six patients suffered acute graft rejection, with the anti-acute rejection treatment of ATG or ALG or methylprednisolone for 3 or 5 days (Table 1). The blood concentrations of maintenance immunosuppressant (Tac, CsA) used in all patients before infection was within the normal reference range at the latest outpatient visit.

**Table 1.** The date of operation, admission and transfer to ICU; the therapy of anti-acute injection and the dosage of TMP-SMX; improvement of the PaO2/FiO2

No	Gender	Induction	Maintenance	Acute rejection therapy	Oxygen therapy	TMP-SMX	PaO2/FiO2
Patient 1	female	ALG	Tac+MPA+Pre	ATG 50 mg×3d	MV	240/1200 mg qid	improved
Patient 2	male	ATG	Tac+MPA+Pre	ATG 50 mg×2d; ALG 0.5 g × 3d	MV	320/1600 mg qid	improved
Patient 3	female	Basiliximab	Tac+MPA+Pre	ATG 25 mg×3d; ATG 25 mg×5d	MV	160/800 mg qid	improved
Patient 4	male	ATG	CsA+MPA+Pre	ATG 50 mg×3d; ATG 25 mg×5d	HFNC	160/800 mg qid	improved
Patient 5	male	ALG	Tac+MPA+Pre	MP 0.5 g × 3d	MV	240/1200 mg qid	improved
Patient 6	male	ATG	Tac+MPA+Pre		MV	240/1200 mg qid	improved
Patient 7	male	ATG	Tac+MPA+Pre	ALG 0.5 g qd×5d; MP 0.5 g × 3d; 0.25 g × 2d;	MV	240/1200 mg qid	improved

ALG: Anti-human T lymphocyte porcine immunoglobulin; ATG: anti-thymocyte globulin; TaC: tacrolimus; MPA: mycophenolic acid; Pre: prednisone; TMP-SMX: trimethoprim–sulfamethoxazole, MP: methylprednisolone; MV, mechanical ventilation; HFNC, high-flow nasal cannula.

## Occurrence of PJP

**Figure 1.**
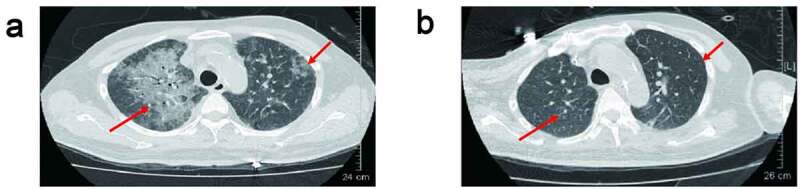
(a) Chest CT of the patient revealed bilateral ground-glass opacity before transferred to our department. (b) Chest CT of the patient revealed bilateral ground-glass opacity absorbed after treatment

Acute rejection occurred within 1 to 6 months after surgery. Two of them occurred acute rejection within 1 month after surgery. Three of them suffered one episode rejection and the other three had two episodes of acute rejection before PJP infection. Five patients developed infection within one month after latest rejection; the other one patient developed infection within six months after acute rejection. All the seven patients developed PJP infection within 4 to 8 months after kidney transplantation. The detailed information is summarized in Table 1. These patients were admitted with fever, cough, or elevated creatinine. The chest CT scan taken before being transferred to our department showed the presence of diffuse, bilateral interstitial infiltrates (ground-glass opacity) in both lungs (Figure 1a). Based on clinical manifestations and imaging characteristics, PJP is suspected clinically.

## Diagnosis and treatment of PJP

**Figure 2.**
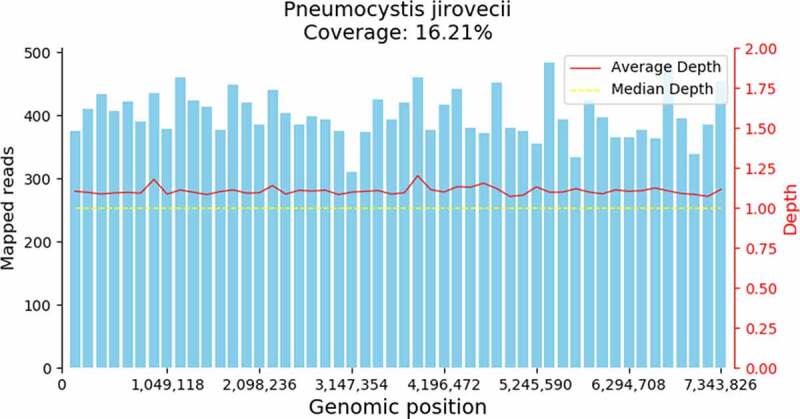
*Pneumocystis jirovecii* genome coverage map of Patient 6

All patients were transferred to our department for short of breath with hypoxemia. Since patients were transferred to our department, they were treated with oral TMP-SMX (160/800 mg to 320/1600 mg, qid) combined with intravenous carpofungin (loading dosage 70 mg on the first day, 50 mg per day from second day). Meanwhile, all oral immunosuppressive drugs were discontinued. And all patients met criteria of respiratory failure [PaO2 less than 70 mmHg or (A-a) DO2 more than 35 mmHg], methylprednisolone (MP) (40 mg, every 12 hours) was given. The Immunofluorescent staining of *P. jirovecii* in sputum or BALF of all patients was negative. The BDG was positive in only two patents. *P. jirovecii* genome (GCF_001477535.1) was detected in all the seven patients (Figure 2 **and** Table 2).

**Table 2.** The NGS sequences of PJP and CMV in blood and BALF, the complication and other infections of the seven patients

No	PJP-BALF	PJP-Blood	CMV-BALF	CMV-Blood	BDG (pg/ml) (reference range 0–10)	Heart failure	CRRT	Complications	Other infections
1	1954	76	21	84	<3.826		yes	HLH	
2	83	Neg	Neg	Neg	14.798	yes	yes	Cerebral infarction	Lung: PDRAB, klebsiella pneumoniae, Candida glabrata UTI: PDRAB
3	1067	Neg	2	Neg	5.888			Pulmonary hemorrhage	Lung: aspergillus, Candida glabrata, Candida subglabrata UTI: Candida subglabrata
4	Not detected	3	Not detected	4	<3.826	yes		Leukopenia	
5	5243	74	198	59	<3.826		yes	Pneumomediastinu subcutaneous emphysema; Gastrointestinal bleeding; Leukopenia; Thrombocytopenia	
6	5177	Neg	Neg	Neg	17.782			Leukopenia; Thrombocytopenia; Increasing ALT; Bilirubinemia	Lung: proteus mirabilis
7	2311	24	Neg	Neg	<3.826		yes	Pneumomediastinu subcutaneous emphysema; Gastrointestinal bleeding; Leukopenia; Thrombocytopenia	Bacterial pneumonia

HLH: Hemophagocytic lymphohistiocytosis; UTI: Urinary tract infection; ALT: Alanine aminotransferase; PDRAB: Pan-drug resistance Acinetobacter baumannii.

Patient 4 received high-flow nasal cannula (HFNC) oxygen therapy, and only mNGS of blood was performed in this patient and *P. jirovecii* sequence was detected in the blood. The other six patients were received mechanical ventilation (MV), mNGS both of BALF and blood were performed by mNGS. Among them, *P. jirovecii* sequence was detected in all the six patients in BALF, but *P. jirovecii* sequence was detected in blood only three patients (Table 2).

In terms of co-infection, the PCR method was used to evaluate the plasma loads of CMV-DNA, and a cutoff value of ≥500 copies/ml was considered positive for CMV infection. The copy number of CMV-DNA in all patients was less than 500 copies/ml. Three of the patients did not detect any sequence of CMV both in BALF and blood, and the other four patients only detected few sequences by mNGS (Table 2).

## Outcome and complications of PJP

With the therapy TMP-SMX combined with caspofungin and MF for the PJP treatment, all patients improved their symptoms. And the oxygenation index (PaO2/FiO2) was improved and infiltrations were absorbed (Figure 1b **and** Table 1). Except patient 4 who received HFNC therapy, the other six patients were treated with MV. Endotracheal tube was successfully removed in five of them (except patient 5 who had not met the standard of weaning from the ventilator), but four of the five patients who successfully removed endotracheal tube received MV again for the complications (patients 1, 2, 3 and 7). Patients 1, 2 and 3 had been transferred back to department of kidney transplantation for a time.

**Figure 3.**
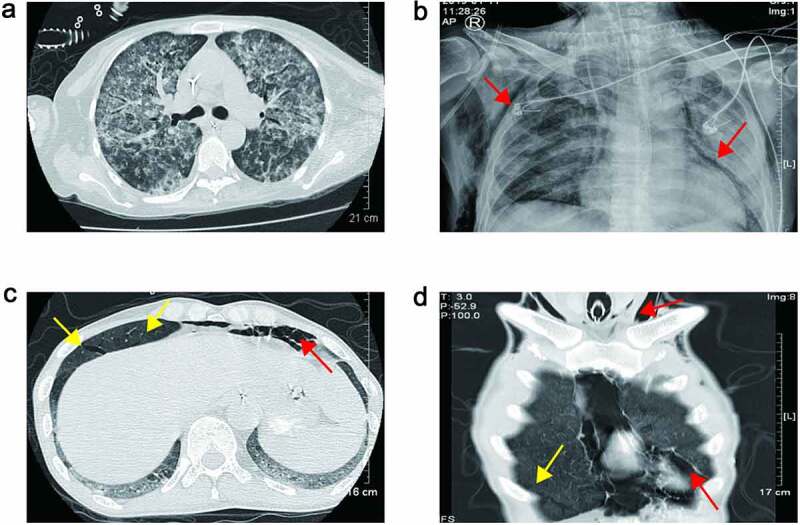
(a) Chest CT scan revealed bilateral diffuse infiltration indicated pulmonary hemorrhage of Patient 3; (b) chest plain radiograph showed pneumomediastinum and subcutaneous emphysema of Patient 5 (red arrow). (c) and (d) Chest CT scan showed pneumomediastinum, subcutaneous emphysema (red arrow) and parenchymal tears of Patient 7 (yellow arrow)

Creatinine levels were elevated in all patients. Acute heart failure occurred in two cases, and four cases received continuous renal replacement therapy (CRRT) (Table 2). Four patients developed bone marrow suppression-leukopenia or thrombocytopenia. After platelet transfusion and drug therapy, they could return to normal without reducing the dosage of TMP-SMX. Patient 6 developed bilirubinemia and elevated alanine aminotransferase. As the dose of TMP-SMX decreased, he gradually returned to normal. Patient 1 developed hemophagocytic lymphohistiocytosis (HLH), patient 2 suffered a cerebral infarction, and patient 3 developed pulmonary hemorrhage with aspergillus pneumonia (Figure 3a **and** Table 2). Both patients 5 and 7 developed pneumomediastinum, subcutaneous emphysema, and gastrointestinal bleeding (Figure 3b**-D and** Table 2). Patient 5 received an emergency cervical skin incision by thoracic surgeon for pneumomediastinum and subcutaneous emphysema, but patient 7 received conservative treatment. In patient 5, gastrointestinal bleeding was not cured with proton pump inhibitor, octreotide and transfusion of red blood cells. Patient 7 suffered hypercalcemia with peak level of 3.41 mmol/L and high plasma parathyroid hormone (PTH) with level of 830.78 pg/ml, he received CRRT to treat hypercalcemia, and a neck computed tomography (CT) scan showed one enhancing nodule (10 mm in diameter) in the rear of the right lobe of his thyroid gland.

And four of them developed other fungal or bacterial infection (Table 2): Patient 2 suffered pneumonia and urinary tract infection (UTI) with pan-drug resistant Acinetobacter baumannii (PDRAB), and other pneumonia with candida glabrata and klebsiella pneumoniae; Patient 3 other fungal infections of the lung with aspergillus, candida glabrata and candida subglabrata, and UTI with candida subglabrata; Patient 6 suffered proteus mirabilis pneumonia; and Patient 7 developed high fever, right lower pneumonia revealed by chest radiograph, he was treated with colistin and TMP-SMX in other hospitals and successfully removed endotracheal tube; at present, he is still being followed up in the outpatient of kidney transplantation department in our hospital.

Patients 4 and 6 were cured and discharged from department of kidney transplantation. The other five patients were discharged from our department for the severe complitations and the economic reasons.

## Discussion

PJP is one of the most common fatal opportunistic pulmonary diseases in renal transplant recipients [15]. The main clinical manifestations were fever, cough, dyspnea, hypoxemia and other nonspecific symptoms. The clinical diagnosis was mainly based on the history, clinical manifestations and chest CT imaging. Microbiology is still the gold standard for diagnosis for PJP, which was based on consensus guidelines, requiring a positive direct immunofluorescence staining on induced sputum or BALF and/or a positive polymerase chain reaction (PCR) assay on a BALF specimen [16]. The positive rate of direct immunofluorescent staining for *P. jirovecii* in sputum or BAL was low. In this study, seven patients were clinically suspected as PJP, they all had dyspnea, hypoxemia and the chest radiograph showed a diffuse interstitial infiltration. But the immunofluorescence staining on sputum or BALF was all negative. Plasma BDG was the most reliable biomarker for serologic diagnosis of PJP. When cutoff value was 100 pg/mL, sensitivity and specificity of the BDG test for PJP were 89.9% and 71.0%, respectively [17]. Despite being a structural molecule of the *P. jirovecii* cell wall, BDG is not a species-specific marker, instead presenting a panfungal character. In this paper, only 2 of seven were positive, and the value was below 100 pg/ml. The mNGS is a powerful tool for the detection and identification of pathogens directly from the specimen [18]. And *P. jirovecii* sequence was detected in all the seven patients. For six of them, both BALF and blood were performed by mNGS, *P. jirovecii* sequence was detected in all the six patients in BALF, but only in three patients *P. jirovecii* sequence was detected in blood. The BALF was firstly recommended to detect for the *P. jirovecii* by mNGS.

CMV infection, allograft rejection, immunosuppressant agents, and a low lymphocyte count have been proposed as risk factors for PJP in kidney transplant recipients (KTRs) after post transplantation prophylaxis [19–21]. Acute rejection and CMV infection were significantly associated with PJP development in KTRs after 6 months of trimethoprim–sulfamethoxazole (TMP-SMX) prophylaxis, two-thirds of patients with PJP presented with a history of rejection or CMV infection, the median time interval between rejection or CMV infection and PJP onset was 6 and 9 months, respectively, it suggests that at least 6 to 9-month chemoprophylaxis may be required for PJP prevention in KTRs with rejection or CMV infection [22]. CMV infection and allograft rejection are independent predictors of PJP, targeted prophylaxis in recipients with CMV infection or allograft rejection may reduce the risk of PJP [23]. The Kidney Disease Improving Global Outcome (KDIGO) and Renal Association (RA)/British Transplantation Society (BTS) clinical practice guidelines (CPGs) were strongly recommended [24]. KDIGO recommends all KTRs receive PJP prophylaxis with TMP-SMX 480 mg daily or 960 mg three times weekly for 3–6 months after transplantation. All KTRs receive PJP prophylaxis with daily TMP-SMX for at least 6 weeks during and after treatment for acute rejection [25]. RA/BTS recommends all patients should receive 3–6 months of treatment with TMP-SMX 480 mg daily for PJP following renal transplantation [26]. The American Society of Transplantation recommends anti-*Pneumocystis* prophylaxis for all SOT recipients at least 6 − 12 months post-transplant (TMP-SMX: 480 mg daily or 960 mg three times weekly). For patients with a history of prior PJP infection or chronic CMV disease, lifelong prophylaxis may be indicated [27]. Unfortunately, in this paper, considering the renal damage caused by TMP-SMX, PJP prophylaxis was only routinely prescribed for 3 months after RT and PJP prophylaxis was not routinely prescribed after acute rejection. In this series, all patients’ copies of CMV-DNA were less than 500 copies per ml, three of them did not detect the CMV sequence both in BALF and blood by mNGS, and the other four patients only detected few CMV sequences. Acute rejection is more likely to develop PJP compared with CMV infection.

KTRs with PJP recommended to be treated with high-dose intravenous TMP-SMX, corticosteroids, and a reduction in immunosuppressive medication. Treatment with corticosteroids for KTRs with moderate to severe PJP (as defined by PaO2 *< *70 mmHg in room air or an alveolar gradient of*>*35 mm Hg) was recommended [25]. According to the guideline, since the PJP was suspected, immunosuppressive medication was discontinued, and methylprednisolone was given. Oral or nasal feeding of TMP-SMX was prescribed. Caspofungin was also prescribed for combination treatment [28]. The combination therapy with caspofungin, an antifungal agent that acts on the cyst form of *p. jirovecii* by inhibiting (1, 3) Beta-D-Glucan synthesis, was presented in experimental mouse models [29]. Jin F et al. found high initial plasma BDG concentration may be a predictor of satisfactory caspofungin response to HIV-negative patients with PJP, the choice of combination therapy with caspofungin and TMP/SMX as initial treatment when BDG≥800 pg/ml in moderate to severe HIV negative patients with PJP [30].

With the combination treatment of TMP-SMX, caspofungin and methylprednisolone, all seven patients’ oxygenation and chest radiographs improved. But some of them had severe and fatal complications that led to a poor prognosis. Patient 1 had hemophagocytic lymphohistiocytosis (HLH). Patient 2 had multi-resistant acinetobacter baumannii in the lung and urinary tract system. Patient 3 had fungal infection in lung and urinary tract system, especially aspergillus pneumoniae infection, which led to pulmonary hemorrhage. Patients 5 and 7 had gastrointestinal bleeding, which may be related to the long-term use of methylprednisolone. In patient 5, he had hypercalcemia. Hypercalcemia develops frequently after renal transplantation and is commonly associated with preexisting secondary hyperparathyroidism. Hypercalcemia in conjunction with PJP is being increasingly reported in renal transplant patients. In all the cases, respiratory symptoms were prominent, hypercalcemia was of mild-to-moderate severity, parathyroid hormone concentration was decreased, and 1,25(OH)(2) D levels were extraordinarily or inappropriately high [31]. But in this patient, the parathyroid hormone was not decreased, but very high, and the neck CT revealed an enhanced parathyroid gland. The hypercalcemia was caused by primary hyperparathyroidism. The gastrointestinal bleeding in patient 7 was not only considering the reason for the use of methylprednisolone, but also should pay attention to the primary hyperparathyroidism [32]. Patients 5 and 7 had spontaneous pneumomediastinum and subcutaneous emphysema. Spontaneous pneumomediastinum with subcutaneous emphysema in PJP is rare [33–35]. In this paper, the chest CT scan of patient 7 showed parenchymal tears which was along the pulmonary veins; this fully confirmed to the mechanism of spontaneous pneumomediastinum.

## Conclusion

In conclusion, we use mNGS to diagnose PJP in KTRs. PJP prophylaxis should be prescribed during and after treatment for acute rejection. TMP-SMX is the first-line and effective drug for PJP treatment, but the complications of PJP are always life-threatening and lead to poor prognosis. We should pay attention to these life-threatening complications.

## Highlight

1. *P. jirovecii* sequence were identified in BALF by the mNGS in renal transplantation recipients.
2. TMP-SMX combined with caspofungin can improve the infection symptoms of PJP in renal transplantation recipients.
3. PJP can lead to other complications.
